# Modeling Oral Multispecies Biofilm Recovery After Antibacterial Treatment

**DOI:** 10.1038/s41598-018-37170-w

**Published:** 2019-01-28

**Authors:** Xiaobo Jing, Xiangya Huang, Markus Haapasalo, Ya Shen, Qi Wang

**Affiliations:** 1 Beijing Computational Science Research Center, Beijing, 100193 China; 2 Department of Conservative Dentistry and Endodontics, Guanghua School of Stomatology, Guangdong Province Key Laboratory of Stomatology, Sun Yat-Sen University, 510055 Guangzhou, Guangdong China; 3 Division of Endodontics, Department of Oral Biological and Medical Sciences, Faculty of Dentistry, University of British Columbia, Vancouver, British Columbia V6T 1Z3 Canada; 4 Department of Mathematics, University of South Carolina, Columbia, SC 29208 USA; 5 School of Materials Science and Engineering, Nankai University, Tianjin, 300071 China

## Abstract

Recovery of multispecies oral biofilms is investigated following treatment by chlorhexidine gluconate (CHX), iodine-potassium iodide (IPI) and Sodium hypochlorite (NaOCl) both experimentally and theoretically. Experimentally, biofilms taken from two donors were exposed to the three antibacterial solutions (irrigants), respectively, for 10 minutes. We observe that (a) live bacterial cell ratios decline for a week after the exposure and the trend then reverses beyond the week; after fifteen weeks, live bacterial cell ratios in biofilms fully return to their pretreatment levels; (b) NaOCl is shown as the strongest antibacterial agent for the oral biofilms; (c) multispecies oral biofilms from different donors showed no difference in their susceptibility to all the bacterial solutions. Guided by the experiment, a mathematical model for biofilm dynamics is developed, accounting for multiple bacterial phenotypes, quorum sensing, and growth factor proteins, to describe the nonlinear time evolutionary behavior of the biofilms. The model captures time evolutionary dynamics of biofilms before and after antibacterial treatment very well. It reveals the important role played by quorum sensing molecules and growth factors in biofilm recovery and verifies that the source of biofilms has a minimal effect to their recovery. The model is also applied to describe the state of biofilms of various ages treated respectively by CHX, IPI and NaOCl, taken from different donors. Good agreement with experimental data predicted by the model is obtained as well, confirming its applicability to modeling biofilm dynamics in general.

## Introduction

Success in endodontics is achieved by a combination of host and treatment factors that contribute to the management of infection, prevention and healing of periapical pathosis^1,2^. Instrumentation is important in removing microbes from the root canals, but after mechanical instrumentation alone, canals often remain heavily infected. Instrumentation creates the space necessary for effective irrigation, which plays the key role in further reducing the number of residual microbes. Irrigating solutions with a strong antibacterial effect are necessary. However, currently available irrigants face great challenges in eliminating all the biofilms from the root canals. The biofilms are results of the microbial growth, where dynamic communities of interacting sessile cells are irreversibly attached to a solid substratum as well as next to each other through a network of extracellular polymeric substances (EPS). Microbial communities growing in biofilms are very difficult to be eradicated with antibacterial agents^3,4^. Microorganisms in mature biofilms can be extremely resistant to antibacterial agents for reasons that have yet been fully understood^5,6^.

There are several antibacterial solutions or irrigants available in the market for endodontics currently. Sodium hypochlorite (NaOCl) is the most popular and important irrigating solution^7^. In water, NaOCl ionizes into sodium ion, Na+, and hypochlorite ion, OCl-, establishing equilibrium with hypochlorous acid (HOCl). Hypochlorous acid is responsible for its antibacterial activity; OCl- is less effective than undissolved HOCl. NaOCl is commonly used in concentrations ranging from 0.5% to 6%. It is the only irrigant in endodontics that can dissolve organic tissue, including the organic part of the smear layer. Another irrigant, Chlorhexidine digluconate (CHX), is widely used in disinfection in dentistry because of its antibacterial activity^8–10^. It has gained considerable popularity in endodontics as an irrigating solution and as an intra-canal medicament. However, CHX has no tissue dissolving capability and therefore it cannot replace sodium hypochlorite. CHX is membrane permeable and attacks the bacterial cytoplasmic inner membrane or the yeast plasma membrane. In high concentrations, CHX causes coagulation of intracellular components^11^. One of the reasons for the popularity of CHX is its substantivity (i.e. continued antibacterial effect), which stems from its ability to bind to hard tissue and maintain antibacterial activity. Iodine compounds are among the oldest disinfectants still actively used. They are best known for their use on surfaces, skin, and operation fields. Iodine is less reactive than the chlorine in hypochlorite. However, it kills rapidly and has bactericidal, fungicidal, tuberculocidal, virucidal, and even sporicidal activity^12^. Iodine penetrates rapidly into the microorganisms and causes cell death by attacking proteins, nucleotides, and other key subcellular components of the cell^12,13^.

Oral biofilm recovery after treatment by two different CHX irrigants for 1, 3, 10, minutes respectively was recently studied by Shen *et al*.^14^. Results from the study showed that biofilms started recovering after two weeks, and then fully returned to the pre-treatment level after eight weeks. However, biofilms in that study were grown from plaque bacteria from a single donor and treated with the CHX solution only so that we cannot make any generalizations since it is inconclusive whether the results are only representative for those specific experimental conditions. This study aims to assess the effect of the source of biofilm bacteria and the type of antibacterial agents on biofilm recovery after exposure to different antibacterial agents.

In this paper, we investigated the recovery of multispecies oral biofilms from two donors after CHX, iodine-potassium iodide (IPI) and sodium hypochlorite (NaOCl) treatments, respectively. Before antibacterial treatments, we grew the biofilms for three weeks. After 10 minutes’ treatment by the antibacterial agents, a large number of bacteria in the biofilms continue to die up to one week and the bacterial dying rate reduces beyond the week. The full recovery of the biofilms needs about 15 weeks afterwards. Interestingly, recovery of biofilms from different donors shows similar dynamics after the treatment.

To fully understand the mechanism of biofilm recovery, a quantitative model in the form of dynamical systems is developed, which accounts for the regulation of growth factor proteins and quorum sensing molecules to bacterial growth and EPS regulation in addition to bacterial cell interactions and their response to antibacterial treatment. The model captures time evolutionary dynamics of the biofilms after antibacterial treatment. The model is then applied to another experiment on biofilm’s susceptibility to three antibacterial agents right after antibacterial treatment to confirm its validity in modeling biofilm dynamics for biofilms of various ages right after the treatment^6^. Using the model, we can explore crucial roles played by the EPS, quorum sensing molecules and growth factor proteins on biofilm dynamics before and after the antibacterial treatment. This model uses a simplified description of the oral bacterial biofilm and performs as good as or even better than the multispecies model given in^14^, making it a robust, quantitative model for studying biofilm dynamics.

## Materials and Methods

### Experiment Development

#### Biofilm specimen preparation and treatment

We used sterile hydroxyapatite (HA) discs (Clarkson Chromatography Products, Williamsport, PA) as the biofilm substrate. They were coated with bovine dermal type I collagen (10 μg/mL collagen in 0.012 N HCl in water) (Cohesion, Palo Alto, CA) as described in refs^15,16^. Supragingival and subgingival plaque were collected from 2 adult volunteers, and suspended in brain heart infusion broth (BHI, Becton Dickinson, Sparks, MD, USA). Separate batches of biofilms from each donor were grown. Coated HA disks were placed in the wells of a 24-well tissue culture plate containing 1.80 mL BHI, and 0.2 mL plaque suspension per well and incubated under anaerobic conditions (Bactron300 Shellab Anaerobic Chamber; Sheldon Manufacturing, Inc., Cornelius, OR) at 37 °C for 3 weeks. Fresh medium was changed weekly.

After 3 weeks of anaerobic incubation, specimens were rinsed twice with 1 mL physiological saline for 1 minute and immersed in 1 mL 1% NaOCl, 0.2/0.4% iodine-potassium iodide (IPI), or 2% CHX for 10 minutes. One percent NaOCl was freshly prepared by diluting a 5.25% stock solution (The Clorox Company, Canada) with distilled water, 0.2/0.4% IPI was prepared by mixing 0.2 g iodine (Sigma Chemical Co, St Louis, MO) in 0.4 g potassium iodide (Sigma Chemical Co) and adding distilled water to a 100-mL volume, and 2% CHX was freshly prepared by diluting in sterile water from 20% stock solution (Sigma Chemical Co). Control specimens, after a rinse with saline, were exposed to 1 mL sterile water for 10 minutes.

#### Examination with CLSM

Samples for confocal laser scanning microscopy (CLSM) for viability staining were collected immediately 1, 3, 8, 11 and 15 weeks after being exposure to the medicaments. Specimens tested with saline for the corresponding time periods were used as controls. For all specimens, the fresh BHI broth was changed once a week.

Two biofilm discs were examined per group each time. The biofilms discs were rinsed in 0.85% physiological saline for 1 minute to remove the culture medium prior to CLSM imaging. They were then stained with a bacterial viability stain (LIVE/DEAD *Bac*light Kit; Thermo Fisher Scientific, Waltham, MA) and scanned with CLSM as described previously^14–17^. Three-dimensional volume stacks were constructed with Imaris 7.2 software (Bitplane Inc, St Paul, MN), and the total volume of red (dead bacteria) and green (live bacteria) fluorescence was measured. The proportion of dead bacteria was calculated from the proportion of red fluorescence to the total of green and red fluorescence. The results were analyzed using Univariate ANOVA followed by post-hoc analysis at a significance level of *P* < 0.05.

All experimental methods reported in this paper were carried out in accordance with relevant guidelines and regulations and all experimental protocols were approved by University of British Columbia. We confirm that informed consent was obtained from all subjects.

### Mathematical Model Formulation

#### Model assumptions

The response of biofilms to antibacterial treatment in experiments reveals that the biofilm recovery process after antibacterial treatment is highly nonlinear and sensitive to antibacterial agents applied. A quantitative model would be very useful to describe dynamics in the process and to identify influential factors (or biomarkers) dictating biofilm recovery after the treatment. In this model, we coarse-grain bacteria into two basic types: the live and the dead bacteria. Their volume fractions are denoted as *L* and *D*, respectively. The volume fractions of the EPS and solvent are also non-negligible and are denoted by *E* and *T*, respectively. There exist functional components in the biofilm that affect the growth/decay and dynamics of the biofilm. These include quorum sensing (QS) molecules, growth factor (GF) proteins and antibacterial agents in a minimal set. Since they normally occupy negligibly small volume fractions in the biofilm, their volume fractions are therefore ignored in this model for simplicity. Under these assumptions, we have

1 $$L+D+E+{T}=1.$$

Previous experiments showed that biofilms would be mature at about the third week, and both the previous and recent experimental evidence showed that mature biofilms would undergo a long period to recover after antibacterial treatment. To model the nonlinear process of biofilm recovery, we introduce a functional component named growth factor in this model, to regulate cell proliferation. This is a proxy for perhaps a set of functional proteins. Identifying the growth factor or growth factors would be a challenging experimental endeavor in the future that we strongly recommend through this quantitative study. Here, we assume the concentration of nutrient during the process is constant for simplicity. The concentrations of QS molecules, antibacterial agents and growth factors are denoted as *H*, *A* and *Q*, respectively.

#### Dynamical equations of bacteria

The birth and death of bacteria are dictated by several factors including bacterial proliferation, the natural cell death and the killing by antibacterial agents (*A*). The proliferation of live bacteria (*L*) is limited by nutrient and the carrying capacity (*L*_*max*_) of the environment, and also affected by growth factors (*Q*). In this model, we assume live bacteria proliferate obeying a logistic model, in which the growth rate is regulated by the growth factor while the cell death is governed by the natural cause and the antibacterial killing. We use a causality diagram to show the mechanisms as follows:

2 $$L\mathop{\to }\limits^{({c}_{2},Q,L)}2L,$$

3 $$L\mathop{\to }\limits^{({r}_{bs},{c}_{3}\gamma A)}D,$$

where ${c}_{2}\frac{{Q}^{2}}{{Q}^{2}+{k}_{q}^{2}}$ is the proliferation rate of live cells, *r*_*bs*_ is the natural death rate of live bacteria and *c*_3_*γA* the antibacterial killing rate. The equation for the rate of change of the live bacteria is then given by

4 $$\frac{dL}{dt}={c}_{2}\frac{{Q}^{2}}{{Q}^{2}+{k}_{q}^{2}}(1-\frac{L}{{L}_{max}})L-{r}_{bs}L-{c}_{3}\gamma AL,$$

where, *k*_*q*_ is a constant in the Hill function and *γ* is a relaxation parameter representing the effect of spatial diffusion in the spatially homogeneous system, which is proposed in the Hinson model^18^ as follows:

5 $$\gamma =\frac{1}{T+\frac{E}{{D}_{pr}}}\frac{2(T+E)}{2+(L+D)},$$

*D*_*pr*_ is a parameter representing the relative diffusivity of EPS. In this approximation, spatial diffusion is effectively replaced in a uniform decay in space, i.e, the homogeneous spatial decay is used as a proxy for heterogeneous diffusion in space.

The natural death and killing of live bacteria contributes to the increase in the population of the dead bacteria. The dead bacteria (*D*) can also disintegrate to shed their surface-attached EPS. So, we assume the dead bacteria eventually disintegrate into EPS (*E*) and solvent (*T*):

6 $$D\mathop{\to }\limits^{({r}_{dp},{k}_{13},A)}E+T.$$

By assuming that the antibacterial agent can in fact slows down the degradation of dead cells, we arrive at the following equation for the rate of change in the volume fraction of dead bacteria:

7 $$\frac{dD}{dt}={r}_{bs}L+{c}_{3}\gamma AL-{r}_{dp}\frac{{k}_{13}}{{k}_{13}+A}D,$$

where *r*_*dp*_ is the rate of degradation of dead bacteria in the absence of antibacterial agents, *k*_13_ is a constant. The first two terms are related to the death of live bacteria cells due to the natural cause and antibacterial effect, respectively, while the last term represents the degradation of dead bacteria into EPS and solvent components from cell lysis and antimicrobial effects. Without being treated by antibacterial agents, the dead cell degrades with a constant rate *r*_*dp*_. With the stress imposed by the antibacterial effect on the biofilm, we surmise the degradation of dead cells would be slowed down. This assumption on the degradation of dead cells is based on the assumption that antibacterial treatment disrupts the natural process of a cell cycle, delaying their disintegration into other biofilm components. This seemingly arbitrary assumption is in fact strongly supported by our model calibration in that the other assumptions on the decay rate would make the model less faithful to the experimental data. We therefore believe the assumption made is credible in this model.

#### Reactive equation of EPS production

The production of EPS (*E*) is affected by quorum-sensing molecules (*H*), live bacterial (*L*) and ultimately the carrying capacity of EPS (*E*_*max*_), at which EPS saturates so that its production ceases. The biochemical process can be described by the causality diagram:

8 $$L\mathop{\to }\limits^{(H,D)}L+E,$$

where ${c}_{5}\frac{{H}^{2}}{{H}^{2}+{k}_{9}^{2}}(1-\frac{E}{{E}_{max}})$ is the EPS production rate due to live bacteria and ${r}_{dp}\frac{{k}_{13}}{{k}_{13}+A}D(1-\frac{E}{{E}_{\max }})$ is the conversion rate due to the degradation of dead bacteria. The equation for the rate of change of EPS volume fraction is then given as follows:

9 $$\frac{dE}{dt}=({c}_{5}L\frac{{H}^{2}}{{H}^{2}+{k}_{9}^{2}}+{r}_{dp}\frac{{k}_{13}}{{k}_{13}+A}D)(1-\frac{E}{{E}_{max}}),$$

where ${k}_{9}$ is a constant in the Hill function for the QS concentration dominated production rate.

#### Dynamical equations of the functional components

The natural degradation of the antibacterial agents, loss in effectiveness, their diffusion and reaction with cells are considered in this quantitative model. The degradation process of antibacterial agents is modeled like this:

10 $$\frac{dA}{dt}=-{c}_{8}AL-{r}_{a}A,$$

where *c*_8_ is the killing rate of the bacteria by antibacterial agents and *r*_*a*_ is the degradation of antibacterial agents in the solution.

The production of QS molecules (*H*) and growth factors (*Q*) are related to the volume fraction of live bacteria (*L*) while, in the meantime, can saturate at their carrying capacities *H*_*max*_ and *Q*_*max*_. For simplicity, we assume the increase in QS molecules (*H*) is also regulated by the growth factor (*Q*) in the form of a Hill function in this model. The mechanisms are summarized as follows:

11 $$L\mathop{\to }\limits^{({c}_{a},Q,L,H)}L+H,$$

12 $$L\mathop{\to }\limits^{({c}_{q},L,Q)}L+Q,$$

where *c*_*a*_, *c*_*q*_ are pre-factors for the QS molecules and growth factors, respectively. We propose the following reactive equations to quantify the mechanisms:

13 $$\frac{dH}{dt}={c}_{a}\frac{{Q}^{2}}{{Q}^{2}+{k}_{q}^{2}}L(1-\frac{H}{{H}_{max}}),$$

14 $$\frac{dQ}{dt}={c}_{q}L(1-\frac{Q}{{Q}_{max}}),$$

where *H*_*max*_, *Q*_*max*_ are carrying capacities for QS molecules and growth factors, respectively.

#### Summary of the governing equations

The coupled dynamical equations of the biofilm in its dimensionless form are summarized as follows

15 $$\frac{dL}{dt}={c}_{2}\frac{{Q}^{2}}{{Q}^{2}+{k}_{q}^{2}}(1-\frac{L}{{L}_{max}})L-{r}_{bs}L-{c}_{3}\gamma AL,$$

16 $$\frac{dD}{dt}={r}_{bs}L+{c}_{3}\gamma AL-{r}_{dp}\frac{{k}_{13}}{{k}_{13}+A}D,$$

17 $$\frac{dE}{dt}=({c}_{5}L\frac{{H}^{2}}{{H}^{2}+{k}_{9}^{2}}+{r}_{dp}\frac{{k}_{13}}{{k}_{13}+A}D)(1-\frac{E}{{E}_{max}}),$$

18 $$\frac{dA}{dt}=-\,{c}_{8}AL-{r}_{a}A,$$

19 $$\frac{dH}{dt}={c}_{a}\frac{{Q}^{2}}{{Q}^{2}+{k}_{q}^{2}}L(1-\frac{H}{{H}_{max}}),$$

20 $$\frac{dQ}{dt}={c}_{q}L(1-\frac{Q}{{Q}_{max}}),$$

where

21 $$L+D+E+T=1,$$

22 $$\gamma =\frac{1}{T+\frac{E}{{D}_{pr}}}\frac{2(T+E)}{2+(L+D)}.$$

**Figure 1:**
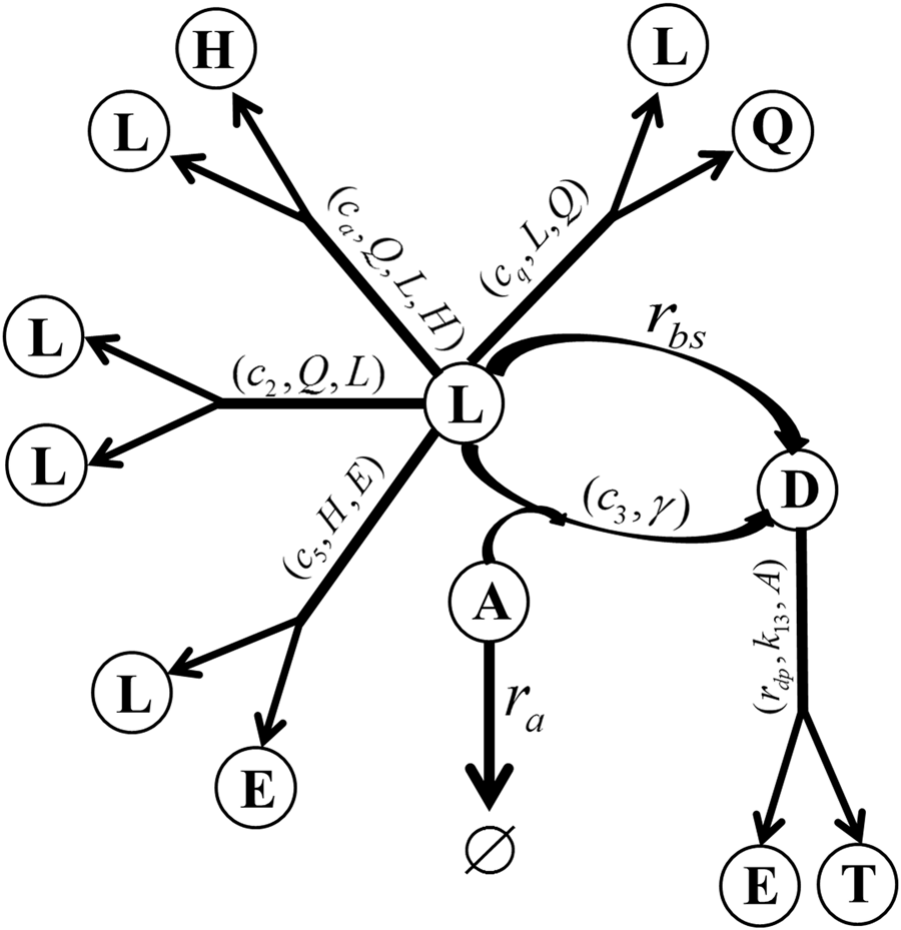
Computational graph of the mathematical model. *L*, *D*, *E*, *T*, *A*, *Q* and *H* are volume fractions or concentrations of live bacteria, dead bacteria, EPS, solvent, antibacterial agents, growth factors and QS molecules, respectively. ∅ Means the degradation of the antibacterial agents. In this directed graph, causal relationships among the components are labelled with arrows and related rates.

The causality relationships used to derive the dynamical equations are summarized in a computational graph in Fig. 1. The nondimensionalization of the equations and variables is given in the supplementary material.

We solve this coupled dynamical system using ode45 solver in Matlab. Some model parameters are taken from the literature while others are calibrated against the experiments reported in this paper and in a previous publication^6^.

### Calibration of Model Parameters

The proposed model given in (15)~(22) is a mechanistic model based on a few fundamental mechanisms and experimentally informed assumptions. Several model parameters need to be calibrated against specific antibacterial agents and biofilm specimen from different donors. We use the following strategy to calibrate the parameters.

1. We first calibrate model parameters independent of antibacterial agents using the control group. Since there is no antibacterial agent in the control group, we set *A*=0. In addition, since the biofilm is fairly mature in the control group, we assume the volume fraction of live bacteria, dead bacteria and EPS in the control group have reached their steady state *L*^*ss*^, *D*^*ss*^, after three weeks. So we set.
  23 $${r}_{bs}{L}^{ss}-{r}_{dp}{D}^{ss}=0.$$
  It follows that
  24 $${r}_{bs}={r}_{dp}\frac{{D}^{ss}}{{L}^{ss}}.$$
  Note that *r*_*dp*_/ (*r*_*bs*_ + *r*_*dp*_) = *L*^*ss*^/(*L*^*ss*^ + *D*^*ss*^) and the live to total cell ratio is measured in the control group. So, we obtain the ratio of *r*_*dp*_ and *r*_*bs*_.
2. We then calibrate model parameters against the control group without the antibacterial effect. The parameters related to antibacterial agents are calibrated the last. After we obtain ratio of *r*_*dp*_ and *r*_*bs*_, we use a bisection method to fit the parameters sequentially. We begin with a prescribed range of parameters resulting from the nondimensionalization and proceed until we get the best fit possible. Specifically, we set an upper limit for each parameter firstly. Then, we use the bisection method to search for the best fit of the model solution to the experimental data for the parameters one-by-one. Before finding the next parameter value using the bisection method, all the already obtained parameter values will be adjusted accordingly as the next parameter is fitted slightly. This method has been used to produce all the parameter values we use throughout this study.

We note that biofilms from different donors may be different. So, their responses to antibacterial treatment can also vary. Once the origin of the biofilm changes, we recalibrate the model parameters. As the result of the model calibration, we find that biofilm recoveries taken from different donors are similar, which indicates that there exist an invariant set of parameter values in the model that are insensitive to donors in biofilm recovery (see Table 1).

**Table 1:** The model parameter values calibrated using the data from the control group and the treated group.

Symbol	Units	Donor 1			Donor 2		
		CHX	IPI	NaOCl	CHX	IPI	NaOCl
H_max_	kg/m^3^	6.59e-04	6.59e-04	6.59e-04	6.59e-04	6.59e-04	6.59e-04
Q_max_	kg/m^3^	2.47e-3	2.47e-3	2.47e-3	2.47e-3	2.47e-3	2.47e-3
c_2_	s^−1^	3e-6	3e-6	3e-6	3e-6	3e-6	3e-6
*c_3_	s^−1^m^3^/kg	2.06e-1	1.5e-1	4.5e-1	1.76e-1	1.40e-1	5.81e-1
c_5_	s^−1^	9.9e-1	9.9e-1	9.9e-1	9.9e-1	9.9e-1	9.9e-1
*c_8_	s^−1^	2e-5	2e-5	9e-6	2e-5	1e-5	1e-5
c_a_	kg/m^3^	8.24e-9	8.24e-9	8.24e-9	8.24e-9	8.24e-9	8.24e-9
c_q_	kg/m^3^	1.65e-8	1.65e-8	1.65e-8	1.65e-8	1.65e-8	1.65e-8
*r_a_	s^−1^	1.8e-7	2e-7	3e-7	8e-8	2.4e-7	2.7e-7
r_bs_	s^−1^	1.6e-7	1.6e-7	1.6e-7	1.8e-7	1.8e-7	1.8e-7
r_dp_	s^−1^	2e-6	2e-6	2e-6	2e-6	2e-6	2e-6
*D_pr_		1.6e-2	5e-2	6e-3	2.66e-2	7e-2	3.5e-3
k_9_	kg/m^3^	6.59e-3	6.59e-3	6.59e-3	6.59e-3	6.59e-3	6.59e-3
*k_13_	kg/m^3^	2.88e-8	1.48e-8	1.48e-8	1.24e-8	1.24e-8	1.48e-8
k_q_	kg/m^3^	2.47e-3	2.47e-3	2.47e-3	2.47e-3	2.47e-3	2.47e-3
L_max_		8e-2	8e-2	8e-2	8e-2	8e-2	8e-2
E_max_		1.2e-2	1.2e-2	1.2e-2	1.2e-2	1.2e-2	1.2e-2
*The leaked agents	kg/m^3^	1.24e-6	9.89e-7	1.3e-7	1.03e-7	1.25e-7	1.43e-7

The starred entries indicate different model parameters for different donors and the others are identical to all donors indicating they are insensitive to different donors. The characteristic time scale *t*_0_ = 1 s and the characteristic concentration scale *C* = 8.24e-3kg/m^3^are used^27^.

As a result, we classify the model parameters into three classes based on parameter calibration results: (I) donor-independent parameters; (II) donor-dependent parameters; (III) antibacterial agent specific parameters. Table 2 lists the parameters in each class. Our study indicates that fluctuations of the parameters in group II on the donors are small. This result supports a single model for all donors’ approach that we are taking in this study.

**Table 2:** Classification of model parameters: (I) donor-independent parameters; (II) donor-dependent parameters; (III) antibacterial agent specific parameters.

Classes	I	II	III
Model parameters	H_max_	c_2_	c_3_
	Q_max_	c_q_	c_8_
	c_5_	r_bs_	r_a_
	c_a_	r_dp_	k_13_
	c_9_	L_max_	D_pr_
	k_q_	E_max_	

## Results

### Experimental results

**Figure 2:**
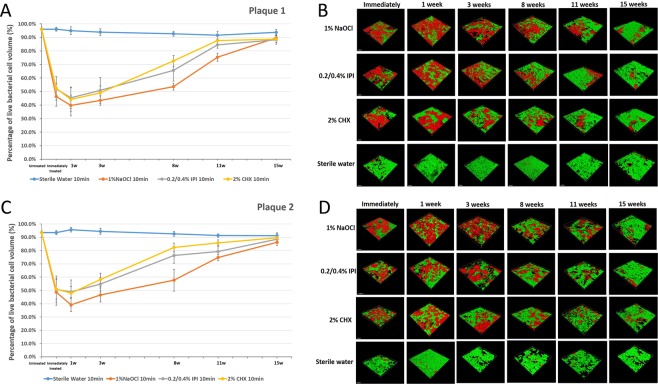
Recovery of bacteria in biofilms taken from donors after 10 min treatment using three antibacterial solutions. The effectiveness of bacterial killing ranks from NaCO1 to IPI to CHX.

A total of 96 HA biofilm discs and 480 scanned areas were analyzed, in which the biofilms were treated for 10 mins by three antibacterial agents three weeks after they were taken from donors. Immediately after treatment, the viability profile of the biofilm population changed, showing an increased number of dead cells (Fig. 2). This occurred in all groups, but was more prominent in biofilms treated with 1% NaOCl. NaOCl showed higher levels of bactericidal activity compared to 2% CHX and 0.2/0.4% IPI (*P* < 0.05; Fig. 2). Cell death in the biofilms continued to intensify for up to one week following exposure to all antibacterial solutions (*P* < 0.001). The viability of the bacterial population increased three weeks after treatment, although biofilms began to recover one week after treatment (Fig. 2). Eight weeks after treatment, the proportion of viable bacteria reached almost that of the pre-treatment level in the CHX group, but less than those of the pre-treatment levels of IPI and NaOCl groups (Fig. 2). Eleven weeks after treatment, bacterial viability in all groups returned to the pre-treatment level (expressed as percentages).

### Numerical Results

**Figure 3:**
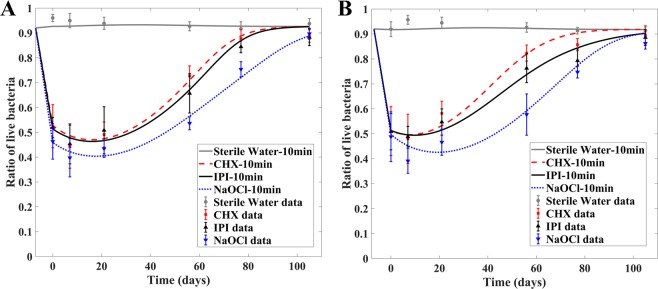
Time evolution of biofilm recovery for donor1 (**A**) and donor2 (**B**). The biofilms were taken from donor 1 and donor 2, respectively, and treated by three antibacterial solutions for 10 mins. At the time of antibacterial treatment, the biofilms had already grown for three weeks. The results indicate that NaOCl is the most effective antibacterial agent among the three and IPI shows slightly higher effectiveness than CHX for both donors. The ratio of live bacteria was calculated using *L/*(*L* + *D*) and measured in the experiments, which are then used in parameter calibration. The smooth curves are the model predictions and the points with error bars are experimentally measured values.

The percentage of live bacteria dropped immediately after antibacterial treatment. Since the bacteria were treated at the end of the third week of biofilm growth, after taken from the donors, and the duration of treatment was 10 mins, we set the moment right after the treatment as time 0 in the dynamical simulations of the model. The percentage recovered to its pretreatment value at the end of the next 15 weeks. The numerical solution of the dynamical model all fall within the error bar of the experimental data, giving a reasonable prediction. As shown in Fig. 3, the ability of NaOCl to kill bacteria is much stronger than that of CHX and IPI. So, the biofilm treated by NaOC1 recovers the most slowly.

**Figure 4:**
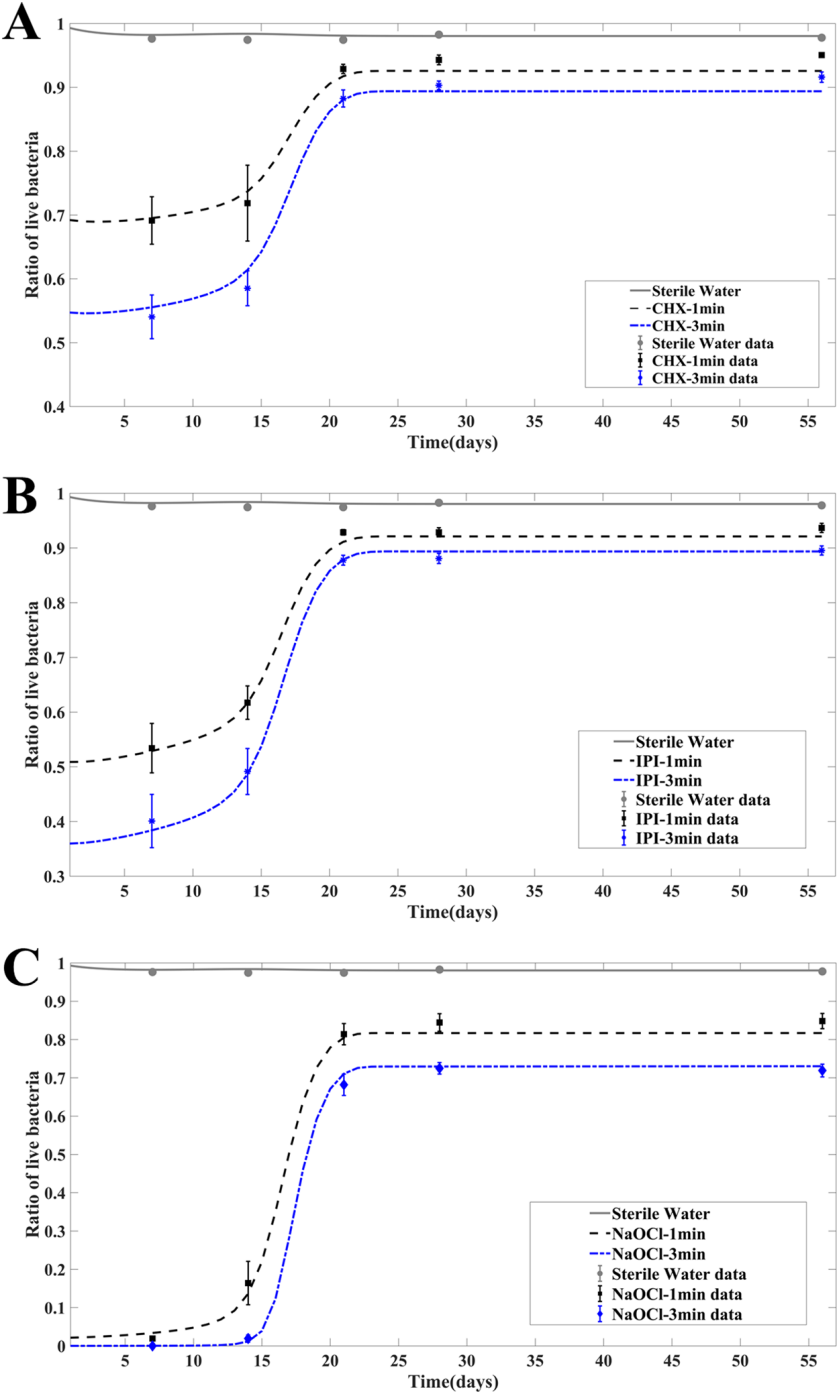
(**A**,**B**) Time evolution of volume fractions of live bacteria (*L*), dead bacteria (*D*), EPS (*E*), concentrations of QS molecules (*H*) and growth factor (*Q*) in the biofilm model without treatment and after 10 min CHX treatment for donor 1 in the experiment. The QS molecule and growth factor level in treated biofilms are lower than those in the control group, shown in (**B**). (**C**,**D**) The same set of selected variables predicted by the model without treatment and right after the 3 min CHX treatment with respect to biofilms of different age groups taken from^6^. The time axis in (**C**,**D**) indicates the age of the biofilm. All the quantities plotted on the curves are the solutions of the model right after the 3 min antibacterial treatment with the initiation condition taken from the control group (i.e., the untreated biofilm). The corresponding solutions from the control group are also plotted for comparison. In (**C**), volume fractions of live, dead bacteria and EPS saturate among the older biofilms for both the treated and the control group. The 3 min treatment induces negligibly small changes in concentrations of QS molecules and growth factor, shown in (**D**). The solutions are plotted beyond the corresponding experimental period (0 to 105 days in subplots (**A**,**B**), 0 to 56 days of age in subplots (**C**,**D**)) to show their asymptotic behavior in time.

In Fig. 4(A,B), we plot the solution of the model prediction for donor 1. We notice that the decline of the live bacterial ratio initially after antibacterial treatment is to a large extent due to the rapid increase in the number of dead bacteria. When the number of dead bacteria starts to decline after a week, the ratio of live bacterial begins to increase as well.

#### Model predictions on responses of biofilms of various ages to antibacterial treatment

**Figure 5:**
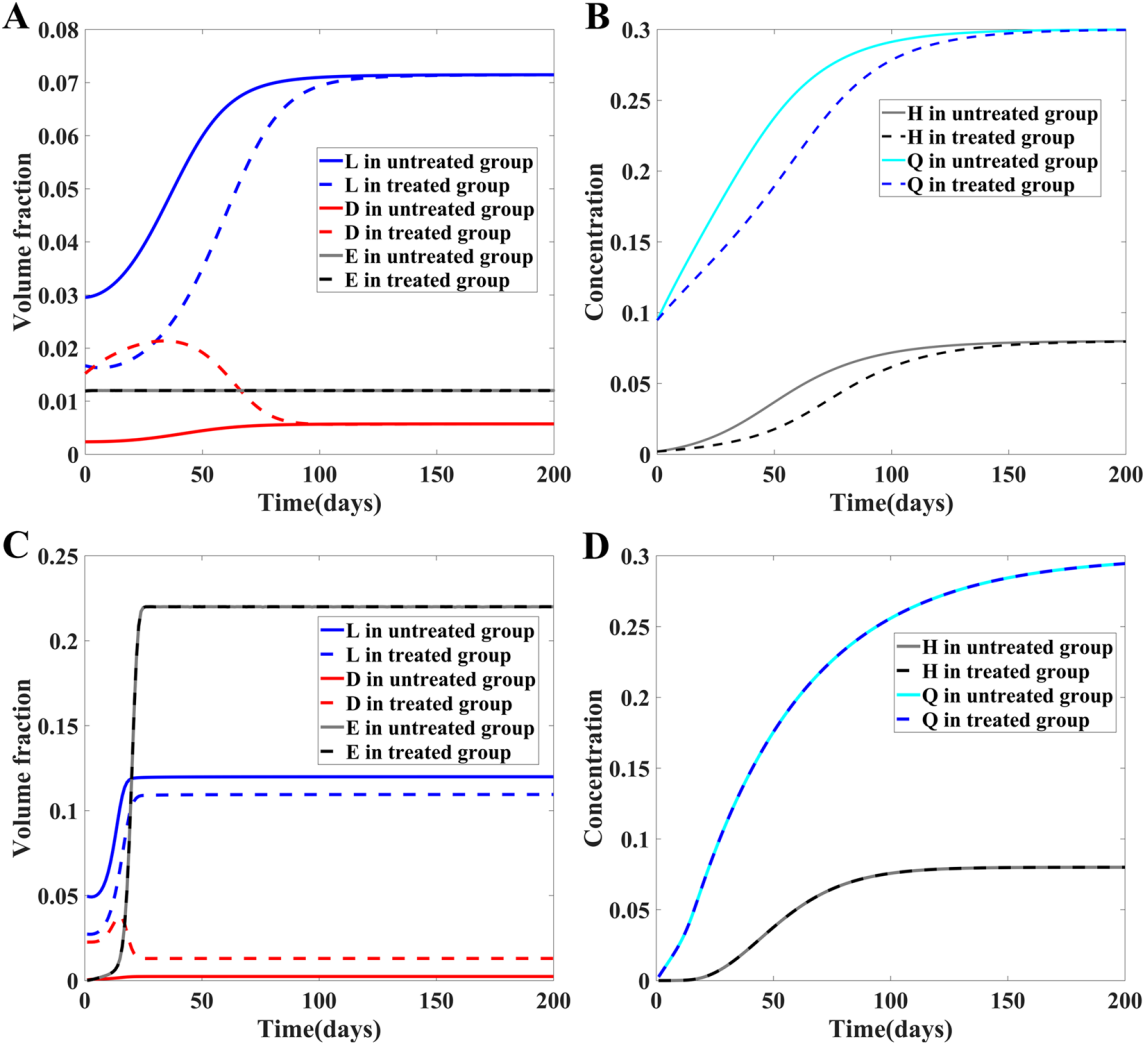
The responses of different aged biofilms to 1 min and 3 min CHX treatment (**A**), 1 min and 3 min IPI treatment (**B**) and 1 min and 3 min NaOCl treatment (**C**), respectively. The time axis here indicates the age of the biofilm. The results presented are the solutions of the quantitative model at different ages right after they are treated by antibacterial agents for 1 min and 3 mins, respectively. For example, the curve plotted at t = 21 days are the solutions of the model at 21days + 1 min (dashed) and 21days + 3 min (dotted) with the antibacterial agent applied to the biofilm at the 21st day for 1 min and 3 mins, respectively.

We apply the mathematical model to another set of experimental data reported by Stojicic *et al*.^6^. Here, we calibrate the model parameters to fit responses of various aged biofilms to the three antibacterial agents following the procedure alluded to earlier. Table 3 lists the parameter values. Stojicic *et al*. investigated the dynamic process of biofilms of various ages varying from the initial attachment of planktonic bacteria to a mature, structurally complex biofilm (0~8 weeks). The biofilms of various ages up to 8 weeks were treated by 2% CHX (Figs 5A), 0.2/0.4% IPI (Fig. 5B) and 1% NaOCl (Fig. 5C) for 1 min and 3 mins, respectively. Their work showed significant difference among the killing ratios of all tested antibacterial solutions in young (less than 2 weeks old) and mature (3 weeks or older) biofilms in all treatments. We use the quantitative model to simulate biofilm growth from the beginning to the moment right after the antibacterial treatment and report the post treatment result. The model fits very well to the experimental data following the parameter calibration procedure alluded to earlier. In addition, we notice that the recalibrated parameter values in the model do not differ much from the other set of parameter values calibrated on a data set of completely different biofilm experiments, indicating the model indeed captures the essence in biofilm growth dynamics. Both experiments and model predictions show that biofilms become mature after about three weeks (Fig. 5). For mature biofilms, the consequence of antibacterial treatment becomes insensitive to the age of the biofilms. For younger biofilms the killing of live bacteria in treated biofilms is significant compared with the mature biofilms (see Fig. 4C). For mature biofilms, we observe that the EPS concentration is high at the time of treatment (Fig. 4C). In the quantitative study, the dimensionless concentration of antibacterial agents we use to briefly treat the biofilms is chosen as 1 and initial volume fraction of live bacteria taken from the donors is chosen as 0.05. The experimental data are taken from the paper^6^.

**Table 3:** The model parameter values calibrated based on the response of different aged biofilms to antibacterial treatment.

Symbol	Units	CHX	IPI	NaOCl
H_max_	kg/m^3^	6.59e-04	6.59e-04	6.59e-04
Q_max_	kg/m^3^	2.47e-3	2.47e-3	2.47e-3
c_2_	s^−1^	3e-4	3e-4	3e-4
*c_3_	s^−1^m^3^/kg	1.20	2.18e-1	8.49
c_5_	s^−1^	9.9e-1	9.9e-1	9.9e-1
*c_8_	s^−1^	1e-2	1e-2	6e-2
c_a_	kg/m^3^	8.24e-9	8.24e-9	8.24e-9
c_q_	kg/m^3^	4.94e-9	4.94e-9	4.94e-9
*r_a_	s^−1^	1.3e-2	1.6e-2	2e-4
r_bs_	s^−1^	1.6e-7	1.6e-7	1.6e-7
r_dp_	s^−1^	5e-6	5e-6	5e-6
*D_pr_		4.8e-2	2.66e-2	1.2e-2
k_9_	kg/m^3^	6.59e-3	6.59e-3	6.59e-3
k_13_	kg/m^3^	1.48e-8	1.48e-8	1.48e-8
k_q_	kg/m^3^	2.47e-3	2.47e-3	2.47e-3
L_max_		1.2e-1	1.2e-1	1.2e-1
E_max_		2.2e-1	2.2e-1	2.2e-1

The starred entries indicate the parameters are sensitive to antibacterial agents in the model while the others are insensitive to antibacterial agents. The characteristic time scale *t*_0_ = 1 s and the characteristic concentration scale *C* = 8.24e-3 kg/m^3^ are used^27^.

#### Role of QS molecules and the growth factor in biofilm dynamics

Since the recovery curves are the same qualitatively for different antibacterial agents and donors, we only show the time evolution of volume fractions of live bacteria (*L*), dead bacteria (*D*), EPS (*E*), the concentration of QS molecules (*H*) and the concentration of the growth factor (*Q*) in biofilms from donor 1 after 10 min CHX treatment as an example. Despite that the percentage of live bacteria continues to decrease as the result of a drastic increase in dead bacteria in the first week after the treatment (Figs 2 and 3), the volume fraction of live bacteria increases along with the growth factor right after the treatment (Fig. 4A,B). In this example, the results show that the EPS volume fraction reaches a plateau before the treatment even begins and is therefore less sensitive to the QS molecule and residual antibacterial agents after the treatment (Fig. 4A,B). As a comparison, we also plot the corresponding variables in the control group, labelled as untreated group. It shows clearly the live and dead bacteria eventually return to the level in the control group, while the QS molecule and the growth factor concentration never fully recover at the end of the simulation.

We also examine time evolution of the aforementioned biofilm components from the responses of different aged biofilms to 3 min CHX treatment (Fig. 4C,D). The residual volume fractions of *L*, *D* and *E* right after the antibacterial treatment vary little for mature biofilms older than 21 days right after treatment, which means that the effectiveness of bacterial killing by the antibacterial agents saturates with respect to the mature biofilms. Despite of the lack of efficiency in killing live bacteria in mature biofilms, the QS molecule and growth factor continues to grow to sustain the recovery of the biofilms. As a comparison, the solutions from the control group are also plotted in Fig. 4C,D. We observe that the 3 min antibacterial treatment does have a sizable impact on the live and dead bacteria for all aged biofilms and the reduction in live bacteria and increase in dead bacteria right after the 3 mins treatment for mature biofilms are indeed insensitive to the age for older biofilms (older than 21 days).

## Discussion

Guided by the experiments, we have developed a quantitative model to describe biofilm recovery after antibacterial treatment. The multispecies oral biofilms grew out of the pooled plaque from two different donors showed nearly no difference in their susceptibility to all antibacterial agents, indicating a lack of variability in sensitivity from different sources of biofilms. The model can fit experimental observations well through a parameter calibration procedure. Although we coarse-grain the bacteria into only two gross types in the model for simplicity, the model can reproduce the recovery process very well for the given experimental data set. Study by Stojicic *et al*.^6^ using multispecies biofilms grown on collagen-coated hydroxyapatite disks from different donors showed that biofilms from 6 different sources had a similar, time-dependent susceptibility pattern. Later, Yang *et al*.^19^. Using an infected dentin model also found that the multispecies biofilms from different donors showed similar susceptibility. The current study shows the same pattern of biofilm recovery after exposure to antibacterial agents in all biofilms taken from different donors. The present study further supports that the source and possible differences in the species composition of the multispecies biofilm have less impact on its susceptibility. To our knowledge, this is the first time the effect of the source of biofilm recovery is investigated. The results clearly indicate that recovery time is less dependent on the type of bacteria present in the biofilm.

We choose NaOCl, CHX, and IPI for the study because they are common endodontic irrigants with different antibacterial mechanisms. NaOCl attacks heat shock proteins in microbes causing the bacteria to form clumps and to die eventually^20^. CHX attacks bacterial cellular membrane^21^ and causes the precipitation of the cytoplasmic contents^22^. The antibacterial mechanism of IPI involves multiple cellular effects by binding to proteins, nucleotides, and fatty acids^23^. CHX and IPI are less effective against biofilms than NaOCl. Post-antibiotic effect (PAE) is the continued suppression of bacterial growth after exposure of the bacteria to an antibacterial agent and removal of this agent from the environment^24–26^. Proposed mechanisms by which the PAE occurs include both nonlethal damage induced by the antibacterial agent and a limited persistence of the antibacterial agent at the bacterial binding site. Factors that affect the duration of the PAE include microorganism-antibacterial combination, duration of antibacterial agent’s exposure, bacterial species, culture medium and experimental conditions. Interestingly, all irrigants have been shown to exhibit extended residual activity after treatment.

The new quantitative model developed in this study simplifies a previous model and even yields an improved fitting to the experimental data^14^. In this model, we group all unknown functional molecules/proteins into a single growth factor, which is a crucially important factor on bacterial growth and dynamics of QS molecules. Meanwhile, we assume dynamics of the growth factor is governed by the live bacteria and limited by its carrying capacity *Q*_*max*_ in the model. The treatment of biofilms by the antibacterial agents does not show any significant effect on the growth factor, except that its growth right after the treatment is much slower than that at a later time. Interestingly, the tamed growth in the growth factor during the first week still fuels the *volume fraction* of live bacteria to increase rather than decrease despite that its absolute population is low. The population of dead bacteria increases initially after the treatment as expected. The overall ratio of live vs total bacteria predicted by the model matches the experimental data very well. The model fitting the recovery curves demonstrates the key role played by the growth factor on time evolution of the biofilms. Although QS molecules remain a steady increase after the treatment, they don’t have any significant effect on the volume fraction of EPS in the biofilm overall. We have also experimented with other modeling approaches without the direct impact of the growth factor to bacterial growth without a success, which adds credibility to our current modeling approach.

The mathematical model is used to describe biofilms from various donors and being treated by various antibacterial solutions. Its sensitivity on model parameters is therefore very important. We combine the control group and treated biofilm data to develop a general parameter calibration protocol, by which we classify the model parameters into three classes: (I) donor-independent parameters: (II) donor-dependent parameters; (III) antibacterial agent specific parameters. We have demonstrated in this study that the model prediction fits quite well to the experimental data. So the parameters in class (I) have no relations with the donors and the antibacterial agents. They are constants in the model. Among the donor and antibacterial agent dependent parameters, our study shows that their fluctuations with respect to different donors and antibacterial agents are minimal. When the model is applied to a completely different data set from a previous experiment for studying biofilm dynamics right after antibacterial treatment of different aged biofilms, good results are obtained as well. For a mechanistic model for such a complex biological system, we believe that this model indeed captures the essence of the biofilm dynamics and can shed insight on investigating details in biofilm dynamics.

## Supplementary information

Apendix
