# Supplementary material for: Modeling Oral Multispecies Biofilm Recovery After Antibacterial Treatment
Source: Sci Rep. 2019 Jan 28;9:804. doi: 10.1038/s41598-018-37170-w (PMC6349915; doi:10.1038/s41598-018-37170-w)
Supplement: Supplementary file 1 — Apendix [file 41598_2018_37170_MOESM1_ESM.pdf]

Modeling Oral Multispecies Biofilm Recovery After Antibacterial Treatment

Xiaobo Jing[1#], Xiangya Huang[2,3#], Markus Haapasalo[3], Ya Shen[3*] and Qi Wang[1,4,5*]

[1]Beijing Computational Science Research Center, Beijing 100193, China;

[2]Department of Conservative Dentistry and Endodontics, Guanghua School of Stomatology, Guangdong Province Key Laboratory of Stomatology, Sun Yat-Sen University, 510055 Guangzhou, Guangdong, China;

[3]Division of Endodontics, Department of Oral Biological and Medical Sciences, Faculty of Dentistry, University of British Columbia, Vancouver, British Columbia, V6T 1Z3 Canada;

[4]Department of Mathematics, University of South Carolina, Columbia, SC 29208, USA;

[5]School of Materials Science and Engineering, Nankai University, Tianjin 300071, China.

[#]These authors contributed equally to this work.

Corresponding author: Prof. Qi Wang (email: qwang@math.sc.edu)
Prof. Ya Shen (email: yashen@dentistry.ubc.ca)

Appendix: Nondimensionalization

Notice that *L*, *D*, *E*, *T* are dimensionless variables while *A*, *H*, *Q* are not. We need to nondimensionalize the dimensional variables and equations in order to analyze and compute the equations. We denote the characteristic time scale as t_0, the common characteristic concentration as C We choose *C*=8.24e-3 kg/m^3 [19] and t_0=1/s. We define the dimensionless parameters as follows:
$\hat{t} \triangleq \frac{t}{t_0}$, $\widehat{c_2} \triangleq c_2 t_0$, $\widehat{k_q} \triangleq \frac{k_q}{C}$, $\widehat{r_{bs}} \triangleq r_{bs} t_0$, $\widehat{c_3} \triangleq c_3 t_0 C$, $\widehat{r_{dp}} \triangleq r_{dp} t_0$, $\widehat{k_{13}} \triangleq \frac{k_{13}}{C}$, $\widehat{c_5} \triangleq c_5 t_0$, $\widehat{k_9} \triangleq \frac{k_9}{C}$, $\hat{A} \triangleq \frac{A}{C}$, $\widehat{c_8} \triangleq c_8 t_0$, $\widehat{r_a} \triangleq r_a t_0$, $\hat{H} \triangleq \frac{H}{C}$, $\hat{Q} \triangleq \frac{Q}{C}$, $\widehat{H_{max}} \triangleq \frac{H_{max}}{C}$, $\widehat{Q_{max}} \triangleq \frac{Q_{max}}{C}$, $\widehat{c_a} \triangleq \frac{c_a}{C} t_0$, $\widehat{k_q} \triangleq \frac{k_q}{C}$, and $\widehat{c_q} \triangleq \frac{c_q}{C} t_0$.
Substituting these parameters into the equations (1)~(6), we obtain the dimensionless equations. If we drop the hat on the variables and the parameters, we recover the dimensionless equations in exactly the same form as the dimensional equations.

$$\frac{dL}{d\hat{t}} = \widehat{c_2} \frac{\hat{Q}^2}{\hat{Q}^2 + k_q^2} (1 - \frac{L}{L_{max}}) L - \widehat{r_{bs}} L - \widehat{c_3} \gamma \hat{A} L, \tag{1}$$

$$\frac{dD}{d\hat{t}} = \widehat{r_{bs}} L + \widehat{c_3} \gamma \hat{A} L - \widehat{r_{dp}} \frac{\widehat{k_{13}}}{\widehat{k_{13}} + \hat{A}} D, \tag{2}$$

$$\frac{dE}{d\hat{t}} = (\widehat{c_5} L \frac{\hat{H}^2}{\hat{H}^2 + \widehat{k_9}^2} + \widehat{r_{dp}} \frac{\widehat{k_{13}}}{\widehat{k_{13}} + \hat{A}} D) \left(1 - \frac{E}{E_{max}}\right), \tag{3}$$

$$\frac{dA}{d\hat{t}} = -\widehat{c_8} \hat{A} L - \widehat{r_a} \hat{A}, \tag{4}$$

$$\frac{dH}{d\hat{t}} = \widehat{c_a} \frac{\hat{Q}^2}{\hat{Q}^2 + \widehat{k_q}^2} L \left(1 - \frac{\hat{H}}{\widehat{H_{max}}}\right), \tag{5}$$

$$\frac{d\hat{Q}}{d\hat{t}} = \widehat{c_q} L \left(1 - \frac{\hat{Q}}{\widehat{Q_{max}}}\right), \tag{6}$$

where

$$L + D + E + T = 1, \tag{7}$$

$$\gamma = \frac{1}{T + \frac{E}{D_{pr}}} \frac{2(T + E)}{2 + (L + D)}. \tag{8}$$

After fitting the parameters from the dimensionless model, we can get the corresponding value in the experiment by perform the transform:
$t \triangleq \hat{t} t_0$, $c_2 \triangleq \widehat{c_2}/t_0$, $k_q \triangleq C\widehat{k_q}$, $r_{bs} \triangleq \widehat{r_{bs}}/t_0$, $c_3 \triangleq \widehat{c_3}/(t_0 C)$, $r_{dp} \triangleq \widehat{r_{dp}}/t_0$, $k_{13} \triangleq C\widehat{k_{13}}$, $c_5 \triangleq \widehat{c_5}/t_0$, $k_9 \triangleq C\widehat{k_9}$, $A \triangleq C\hat{A}$, $c_8 \triangleq \widehat{c_8}/t_0$, $r_a \triangleq \widehat{r_a}/t_0$, $H \triangleq \hat{H} C$,

$Q \triangleq C\hat{Q}, H_{max} \triangleq C\widehat{H_{max}}$, $Q_{max} \triangleq C\widehat{Q_{max}}$, $c_a \triangleq \widehat{c_a}C/t_0$, $k_q \triangleq C\widehat{k_q}$, and $c_q \triangleq C\widehat{c_q}/ t_0$.
